# Artificial Intelligence–Driven High-Resolution Coronary CT Angiography for PCI Planning in Severe Coronary Calcification

**DOI:** 10.1016/j.jaccas.2025.105319

**Published:** 2025-10-15

**Authors:** Hirofumi Ohashi, Hirohiko Ando, Masanobu Fujimoto, Wataru Suzuki, Koshiro Sakai, Tetsuya Amano

**Affiliations:** a Department of Cardiology, Aichi Medical University, Aichi, Japan; b Department of Medicine, Division of Cardiology, Showa University School of Medicine, Tokyo, Japan

**Keywords:** artificial intelligence, calcified coronary lesion, coronary computed tomography angiography, high-resolution imaging, percutaneous coronary intervention

## Abstract

**Background:**

Severe calcified lesions are difficult to assess using conventional coronary computed tomography angiography (CCTA). Artificial intelligence (AI)–driven reconstruction may help address this limitation.

**Case Summary:**

A 93-year-old man with angina underwent AI-enhanced high-resolution CCTA using the Aquilion ONE/INSIGHT Edition (Canon Medical Systems). The AI-enhanced imaging clearly revealed severe eccentric calcification from the distal left main trunk to the proximal left anterior descending artery. Intravascular ultrasound confirmed this finding. Percutaneous coronary intervention (PCI) was performed with orbital atherectomy, cutting balloon predilation, and drug-eluting stent implantation, achieving good stent expansion.

**Discussion:**

AI-enhanced CCTA enabled the accurate visualization and measurement of calcified plaques, including their arc and thickness, facilitating the selection of appropriate devices and lesion preparation for PCI.

**Take-Home Messages:**

AI-enhanced CCTA provides precise evaluation of severely calcified coronary plaques. It guides PCI strategy by accurately assessing calcium burden and plaque morphology.

## Graphical Abstract

**Figure:**
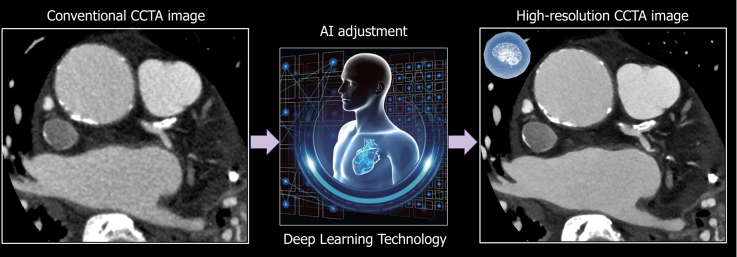

## History of Presentation and Past Medical History

A 93-year-old man presented with Canadian Cardiovascular Society (CCS) class III effort angina. The patient had a history of hypertension, hyperlipidemia, and severe aortic stenosis but no history of myocardial infarction, prior coronary interventions, or stroke.

**Take-Home Messages**

• AI-enhanced high-resolution CCTA provides a precise assessment of severely calcified coronary lesions, enabling optimized lesion preparation for PCI.
• AI-driven imaging technologies are emerging as critical tools for complex coronary intervention planning.

## Differential Diagnosis

Differential diagnoses included stable angina due to coronary atherosclerosis, microvascular angina, and aortic stenosis-related angina.

## Investigations

**Figure 1:**
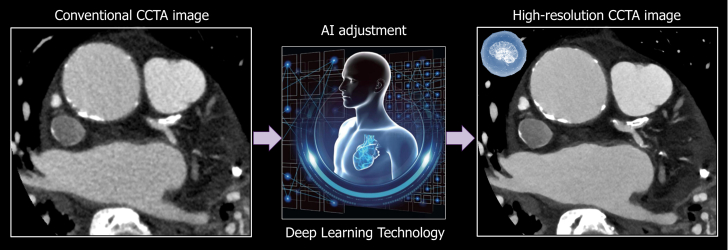
AI-Enhanced High-Resolution CCTA Comparison of conventional CCTA and AI-enhanced CCTA images. AI adjustment using the Precise IQ Engine (PIQE) algorithm resulted in superior spatial resolution. AI = artificial intelligence; CCTA = coronary computed tomography angiography.

**Figure 2:**
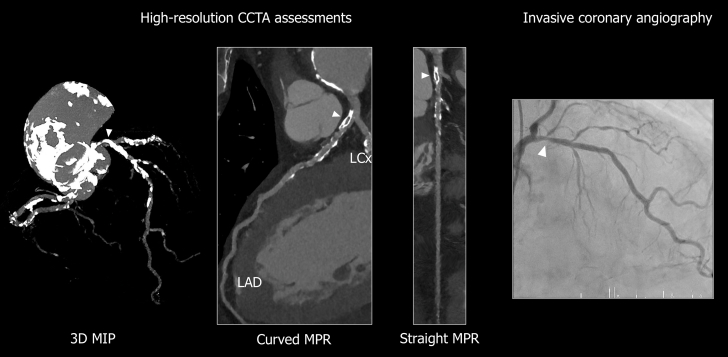
Assessment of CCTA and Invasive Angiography CCTA assessment of the lesion (arrowheads). Severe calcification extending from the distal left main trunk to the mid left anterior descending artery is clearly depicted. Invasive coronary angiography confirmed similar findings. 3D = three-dimensional; CCTA = coronary computed tomography angiography; LAD = left anterior descending artery; LCx = left circumflex artery; MIP = maximum intensity projection; MPR = multiplanar reformation.

**Figure 3:**
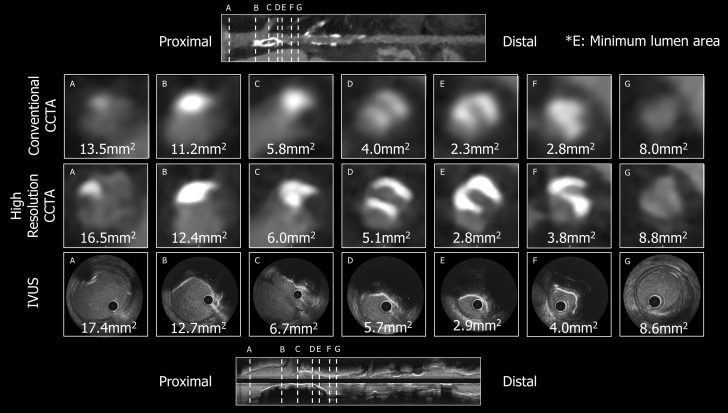
Comparison of Lumen Area and Calcified Plaque by Imaging Modalities Comparison of lumen area and calcified plaque assessment between conventional CCTA, AI-enhanced CCTA, and IVUS. AI-enhanced CCTA provided lumen measurements closer to IVUS (2.8 vs 2.9 mm^2^) compared with conventional CCTA (2.3 mm^2^) and depicted calcified plaque more clearly. AI = artificial intelligence; CCTA = coronary computed tomography angiography; IVUS = intravascular ultrasound.

Coronary computed tomography angiography (CCTA) was performed using the Aquilion ONE/INSIGHT Edition (Canon Medical Systems) with the Precise IQ Engine (PIQE) super-resolution deep-learning reconstruction algorithm, at a heart rate of 64 beats/min and 120 kVp (Figure 1). CCTA demonstrated severe eccentric calcification with a 270° arc extending from the distal left main trunk to the proximal left anterior descending artery (Figure 2). Invasive coronary angiography confirmed moderate stenosis at the proximal left anterior descending artery. Intravascular ultrasound (AltaView, Terumo) demonstrated severe calcification, with a minimum lumen area of 2.9 mm^2^, closely matching the AI-enhanced CCTA measurement (2.8 mm^2^), while conventional CCTA underestimated the lumen area (2.3 mm^2^) (Figure 3). AI-enhanced CCTA provided significantly improved clarity of the calcified plaque morphology compared with traditional CCTA.

## Management

**Figure 4:**
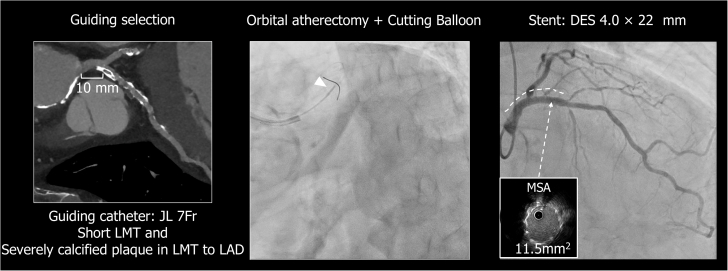
PCI Strategy and Procedural Outcome Images show CT-guided selection of the guiding catheter, the procedural steps of the PCI, and the final procedural result. CT = computed tomography; DES = drug-eluting stent; LAD = left anterior descending artery; LMT = left main trunk; MSA = minimum stent area; PCI = percutaneous coronary intervention.

Percutaneous coronary intervention (PCI) was performed with orbital atherectomy (Diamondback 360, Cardiovascular Systems), followed by cutting balloon (Boston Scientific) predilation. A 4.0 × 22 mm drug-eluting stent (Xience Skypoint, Abbott Vascular) was deployed from the distal left main trunk to the proximal left anterior descending artery. Final intravascular ultrasound confirmed optimal stent expansion, with a minimum stent area of 11.5 mm^2^ (Figure 4).

## Outcome and Follow-Up

The patient recovered uneventfully and was discharged without complications. At follow-up, he remained asymptomatic without evidence of recurrent angina.

## Discussion

Accurate evaluation of calcified coronary lesions is essential for successful PCI results. Conventional CCTA is often limited by blooming artifacts and lower spatial resolution, impairing plaque characterization. AI-enhanced CCTA improves spatial resolution and enables detailed visualization of complex calcified plaques, including the extent and morphology of the calcification.^1^^,^^2^ In this case, AI-enhanced CCTA successfully depicted a calcium plaque component and precise lumen size, which correlated well with findings on intravascular ultrasound, guiding adequate lesion preparation and procedural success. Although limited to a single case, these findings highlight the potential for AI-enhanced imaging to affect decision-making in severe calcified coronary interventions.

## Conclusions

AI-enhanced high-resolution CCTA significantly improves preprocedural assessment of complex calcified coronary lesions, contributing to optimized PCI planning and clinical outcomes.

**Equipment List:** Equipment Used for CT and PCI

CT scanner	Aquilion ONE/INSIGHT Edition (Canon Medical Systems) with Precise IQ Engine (PIQE)
Orbital atherectomy device	Diamondback 360 coronary orbital atherectomy system (Cardiovascular Systems)
Cutting balloon	Wolverine coronary cutting balloon (Boston Scientific)
Drug-eluting stent	Xience Skypoint drug-eluting stent (Abbott Vascular)
Intravascular ultrasound	AltaView intravascular ultrasound catheter (Terumo)
Guiding catheter	Heartrail II guiding catheter (Judkins left 7-F) (Terumo)

CT = computed tomography.

## Funding Support and Author Disclosures

The authors have reported that they have no relationships relevant to the contents of this paper to disclose.
